# Case Report: Combined cataract surgery and goniosynechialysis in elderly patients with iridoschisis—a report of two cases

**DOI:** 10.3389/fmed.2026.1873711

**Published:** 2026-06-29

**Authors:** Shuo Xu, Yinong Zhang, Lingyan Cheng

**Affiliations:** Department of Ophthalmology, Jiangnan University Medical Center, Wuxi, China

**Keywords:** cataract, glaucoma, goniosynechialysis, iridoschisis, phacoemulsification

## Abstract

Iridoschisis, a rare condition involving separation between the anterior iris stroma and deeper layers, primarily occurs in patients between 60 and 70 years of age. The disorder often produces a distinctive “shredded wheat” appearance from iris fibers floating in the anterior chamber, and it commonly coexists with angle-closure glaucoma and cataracts. We present two cases where combined surgical management proved effective. The first case was an 81-year-old woman with bilateral iridoschisis complicated by cataract. Following laser peripheral iridotomy (LPI), gonioscopic examination indicated continued angle closure. Combined phacoemulsification and goniosynechialysis surgery achieved normalized IOP and visual improvement through 18-month follow-up. The second patient was an 82-year-old man with bilateral iridoschisis and narrow angles. The first eye underwent standard cataract surgery. When operating on the second eye, intraoperative gonioscopy identified peripheral anterior synechiae, leading us to add goniosynechialysis. Postoperatively, both eyes showed excellent visual outcomes and stable IOP. For iridoschisis patients, the dual procedure of phacoemulsification and goniosynechialysis addresses two problems simultaneously: the cataract and the anatomical cause of angle closure. The combination thus offers a practical solution for complex iridoschisis cases.

## 1 Introduction

Iridoschisis, a rare ocular condition, involves separation of the anterior iris stroma from the deeper posterior stroma and muscle layers. The posterior iris layer usually remains intact, which maintains sphincter and dilator muscle function (1). These separated stromal fibers are seen floating in the aqueous humor of the anterior chamber, resulting the typical “shredded wheat” appearance (2). While primarily affecting inferior quadrants, iridoschisis may also involve other iris areas (1).

The condition predominantly occurs in the 60–70 year age range, typically involves both eyes, and may rarely appear in younger adults. The genetic basis of iridoschisis is unclear, but the condition most commonly follows trauma or syphilitic infection (3, 4). Patients with iridoschisis typically experience no ocular discomfort, and frequently found during routine eye examinations or investigations for other eye problems. Commonly associated conditions comprise angle-closure glaucoma (the predominant form), narrow angles, cataracts, lens subluxation, keratoconus, bullous keratopathy, syphilitic interstitial keratitis, and microphthalmos (3–7). This report presents two iridoschisis cases where combined phacoemulsification and goniosynechialysis achieved satisfactory therapeutic outcomes.

## 2 Case presentation

### 2.1 Case 1

The first patient was an 81-year-old woman. The patient reported two months of recurrent, recently worsening distending pain in the right eye, accompanied by seven days of blurred vision. The patient had no history of ocular trauma, inherited eye diseases, or previous eye surgery. She was currently under active management for hypertension, diabetes, and vertigo. Examination findings included the preoperative uncorrected visual acuity (UCVA) of 20/200 (right eye) and 20/50 (left eye), the best-corrected visual acuity (BCVA) of 20/40 (right eye) and 20/40 (left eye), with corresponding intraocular pressure (IOP) measurements of 34.2 mmHg OD and 11.7 mmHg OS.

**Figure 1:**
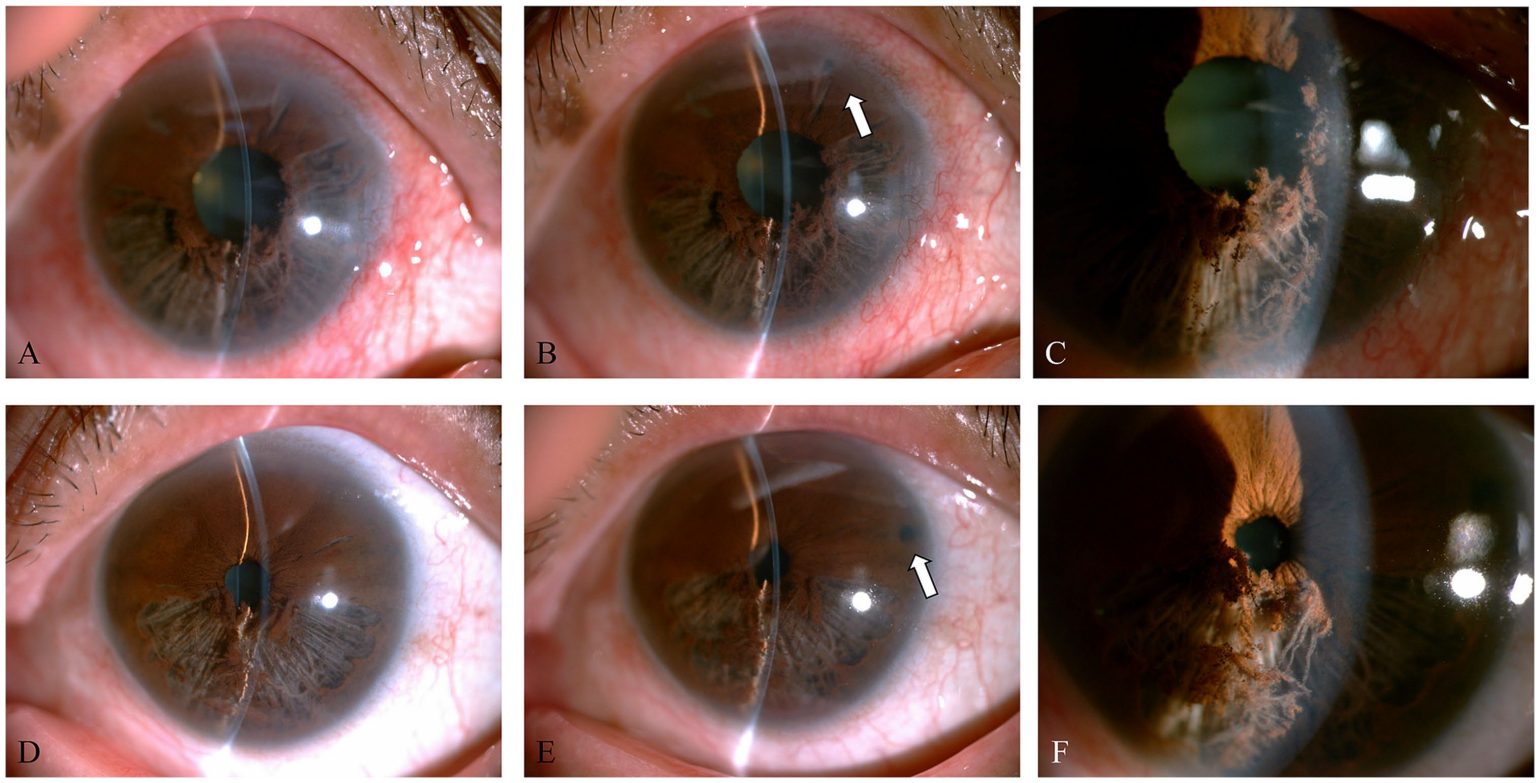
**(A)** Slit lamp examination photographs of the right eye preoperatively. **(B,C)** Photos of the anterior segment of the right eye after laser peripheral iridotomy (LPI). **(D)** Slit lamp examination photographs of the left eye preoperatively. **(E,F)** Photos of the anterior segment of the left eye after LPI. The white arrow points to the LPI cut.

**Figure 2:**
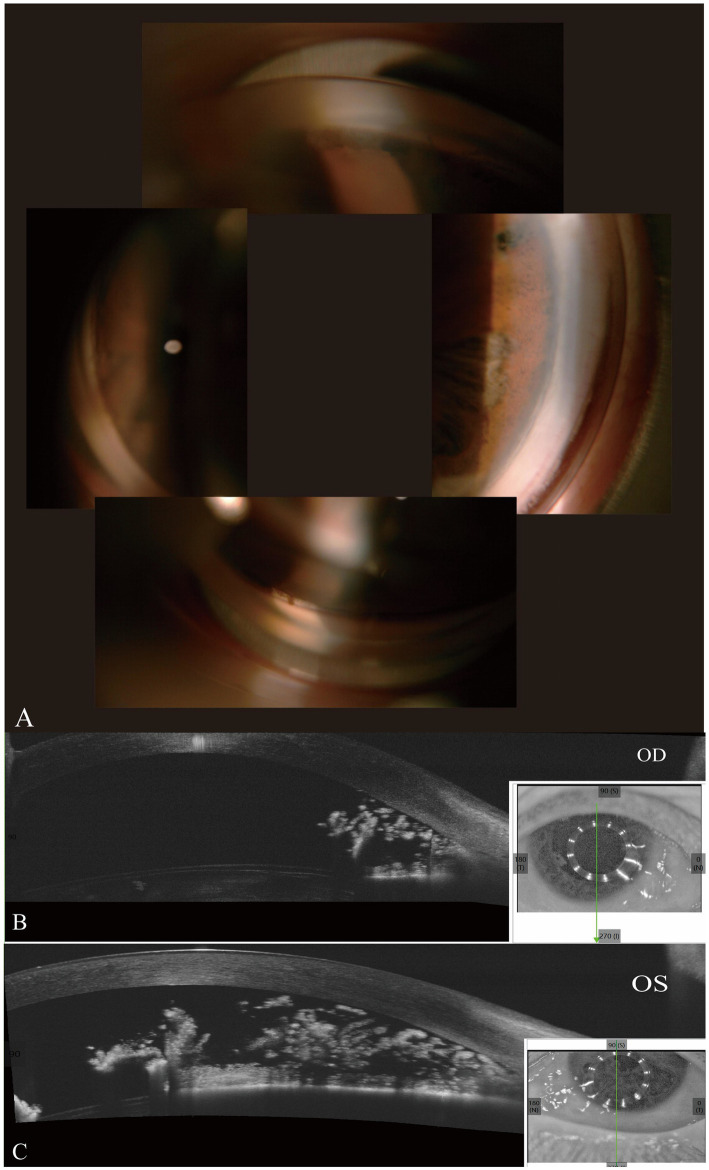
**(A)** Gonioscopic findings of left eye preoperatively. **(B)** Preoperatively AS-OCT image of the right eye. **(C)** Preoperatively AS-OCT image of the left eye.

Slit-lamp examination of the anterior segment showed ciliary congestion and slight corneal edema in the right eye (Figure 1A). Gonioscopic examination revealed a shallow anterior chamber and grade IV angle closure (Scheie classification) circumferentially. No gonioscopic images of the right eye were recorded. Stromal separation of the iris allowed fibers to float into the anterior chamber. Floating iris fibers in the anterior chamber caused segmental iridocorneal contact, with iridoschisis extending from 2 to 9 o’clock in the right eye. The pupil measured about 3.5 mm with sluggish light response. The lens showed nuclear opacities, and fundus images were indistinct. The cup-to-disc (C/D) ratio measured about 0.5. In the left eye, iridoschisis extended from 3 to 9 o’clock positions. The anterior chamber was shallow with an N I angle (Scheie classification; Figure 1D). Gonioscopy revealed circumferential visibility of the functional trabecular meshwork without iridocorneal adhesion. Scant mesodermal remnants were present and exhibited Grade I pigmentation (Figure 2A). The pupil measured about 2.5 mm in diameter. Nuclear opacities in the lens, and the C/D ratio was about 0.4.

Ultrasound biomicroscopy (UBM) showed a closed angle in all quadrants of the right eye and a narrow, slit-like angle opening in the left eye. Anterior segment optical coherence tomography (AS-OCT) revealed iris stromal layer separation in iridoschisis-affected sectors and narrow iridocorneal angles bilaterally (Figures 2B,C). RNFL thinning was evident on fundus OCT, correlating with perimetric visual field defects (Supplementary Figures S1, S2). Corneal endothelial cell density was 2,593 cells/mm^2^ in the right eye (Slit-lamp examination revealed no abnormality in the patient’s left cornea, and as no surgery was planned, corneal endothelial cell count was not performed accordingly). Axial lengths: 22.46 mm OD, 22.05 mm OS. Serological testing showed positive results for treponema pallidum antibody and TRUST (titer 1:1).

Bilateral laser peripheral iridotomy (LPI) was performed on presentation day (Figures 1B,C,E,F), with subsequent admission for right eye surgery. Following preoperative assessment, the patient underwent right eye goniosynechialysis with phacoemulsification and IOL implantation under local anesthesia. The implanted lens was a TECNIS ZA9003 (Abbott Medical Optics) foldable hydrophobic acrylic aspheric monofocal IOL, placed within the capsular bag. Following IOL implantation, gonioscopy confirmed complete angle closure. 1–2 mL of a cohesive viscoelastic agent (AiVei, Bausch & Lomb Freda Shandong Pharmaceutical Co., Ltd.) was introduced into the anterior chamber via the main corneal incision. Blunt goniosynechialysis was then performed using gentle hydraulic pressure, without specialized instrument for goniosynechialysis, successfully opening approximately 270° of the angle (inferior, nasal, and temporal). The procedure was completed without intraoperative complications. Mannitol (250 mL) was administered preoperatively during pupil dilation for glaucoma prophylaxis. Surgical maneuvers included cutting iris fibrils with Vannas capsulotomy scissors.

**Figure 3:**
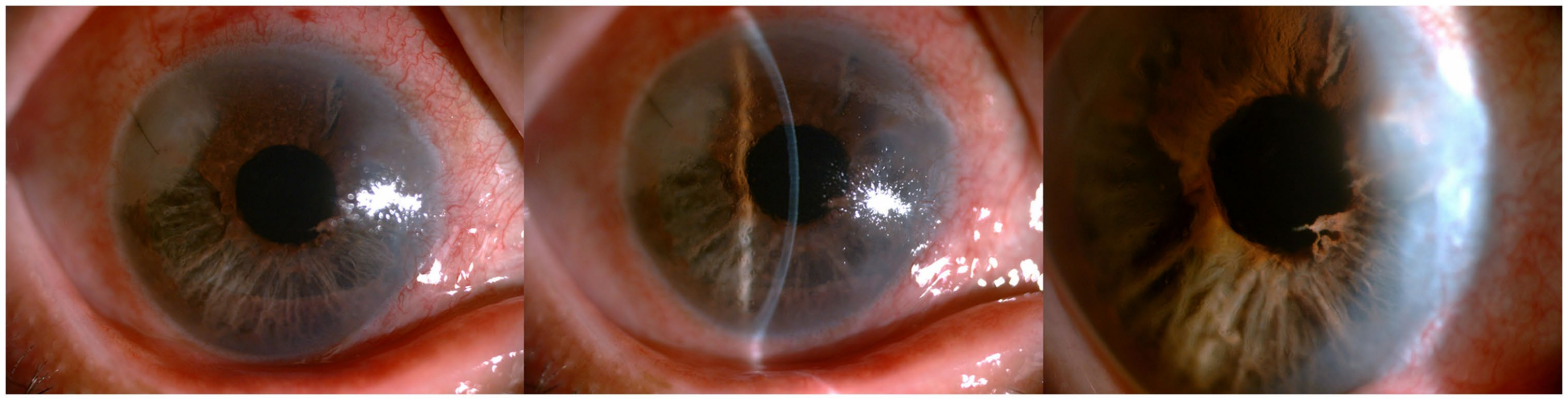
Postsurgical photograph of the anterior segments of right eye.

Postoperatively, the patient was started on a topical combined tobramycin-dexamethasone ointment four times daily. On the first postoperative day, BCVA in the right eye was 20/200, with an IOP of 9.2 mmHg. Slit-lamp examination showed moderate ocular hyperaemia and corneal oedema; the anterior chamber depth was normal, the pupil was round, and the split iris stroma adjacent to the corneal endothelium appeared nearly excised (Figure 3). After the first week, the regimen was switched to tobramycin-dexamethasone eye drops at the same frequency, which was then tapered weekly. At this one-week visit, BCVA had improved to 20/50 with an IOP of 10.1 mmHg. The ocular hyperaemia had resolved, and the corneal oedema showed mild improvement. Gonioscopy showed a functional trabecular meshwork in all quadrants except the superior one. No significant peripheral anterior synechiae or abnormal pigmentation was noted. Throughout the entire follow-up period, IOP remained within normal limits, never requiring additional pressure-lowering medication. At the most recent follow-up (18 months postoperatively), BCVA was 20/40 with an IOP of 11.4 mmHg. With no corneal edema or Descemet’s folds observed on long-term slit-lamp evaluation, postoperative endothelial cell counting was not undertaken.

### 2.2 Case 2

An 82-year-old man reported progressively worsening bilateral blurred vision over a two-year period. There was no history of ocular trauma or inherited ocular conditions. Systemic history included coronary artery disease and atrial fibrillation on current medication. Ocular examination revealed UCVA of 20/40 OD and 20/50 OS, the BCVA of 20/40 OD and 20/25 OS, IOP measured 12.3 mmHg OD and 11.5 mmHg OS.

**Figure 4:**
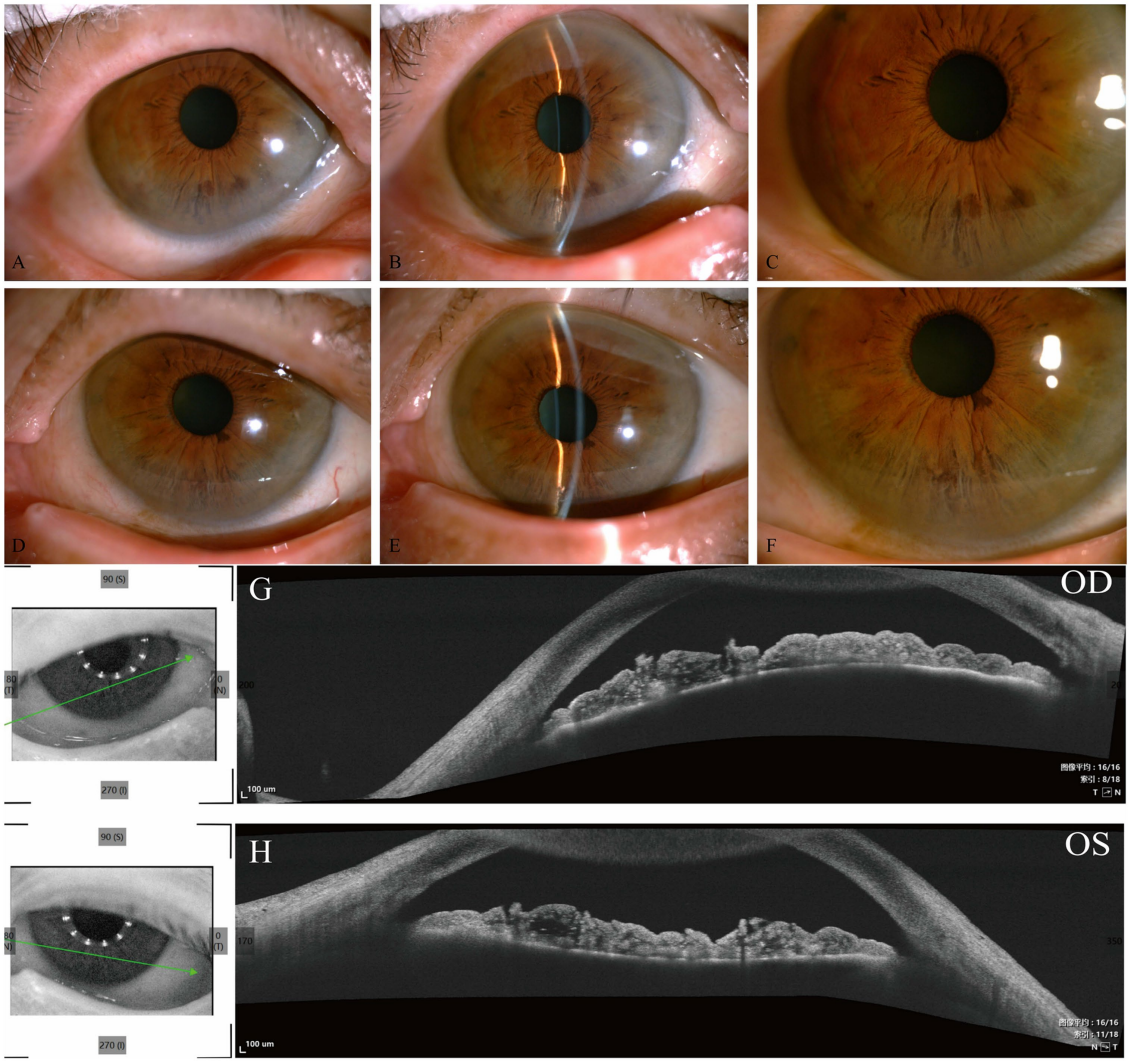
**(A–C)** Slit lamp examination photographs of the right eye preoperatively. **(D–F)** Slit lamp examination photographs of the left eye preoperatively. **(G)** Preoperatively AS-OCT image of the right eye. **(H)** Preoperatively AS-OCT image of the left eye.

Slit-lamp biomicroscopy of the anterior segment showed transparent corneas and shallow anterior chambers bilaterally (Figures 4A–F). Bilateral findings included round pupils with normal light reflexes. The inferior iris was loosened, and cable-like tissue was observed in both eyes. Fundus examination revealed normal findings bilaterally, with no glaucomatous signs detected on OCT (Supplementary Figure S3).

Anterior chamber depth was 2.63 mm in the right eye and 2.74 mm in the left eye, as measured by UBM, with open angles circumferentially. Iridoschisis was evident on AS-OCT (Figures 4G,H). Axial length was 23.27 mm in both eyes. Corneal endothelial cell counts were 2,637 cells/mm^2^ OD and 2,647 cells/mm^2^ OS. Treponema pallidum antibody testing was negative.

The left eye underwent conventional phacoemulsification with TECNIS ZA9003 IOL (Abbott Medical Optics) implantation. Five months later, the right eye received the same procedure. After IOL implantation, gonioscopy confirmed angle closure at the 7–8 o’clock position. Goniosynechialysis was then performed by injecting 1-2 mL of the cohesive viscoelastic agent through the main corneal incision, without specialized instrument for goniosynechialysis, and successfully opened the closed angle.

Topical therapy with combined tobramycin-dexamethasone ointment (four times daily) was initiated postoperatively. The patient’s first-day evaluation revealed a right-eye BCVA of 20/30 and an IOP of 6.8 mmHg, and a left-eye BCVA of 20/100 and an IOP of 7.5 mmHg. In the second week, the ointment was replaced with tobramycin-dexamethasone eye drops at the same dosing frequency; this was then tapered weekly. When reassessed at one week, BCVA in the right eye had improved to 20/25 (IOP 9.1 mmHg), while in the left eye it was 20/25 (IOP 9.5 mmHg). Dynamic gonioscopic examination revealed a circumferentially visible functional trabecular meshwork, with no PAS observed. No additional anti-glaucoma medications were used at any point, as intraocular pressure stayed consistently within normal limits throughout the postoperative period. At the most recent review (10 months after right-eye surgery), visual acuity in the right eye was stable at 20/25 and IOP was 9.4 mmHg; in the left eye, visual acuity was 20/20 with an IOP of 9.9 mmHg. Given the absence of corneal edema on long-term slit-lamp follow-up, postoperative endothelial cell counts were not obtained for these patients.

## 3 Discussion

Schmitt (8) initially noted anterior iris layer detachment. The condition was then formally named by Lowenstein and Foster in 1945, who described deep, parallel stromal splitting (9). The histopathology of iridoschisis features stromal fibrosis and atrophy, leading to interlayer gap formation within the iris (10). The etiology of iridoschisis remains uncertain regarding hereditary versus sporadic origins.

Iridoschisis must be differentiated from other stromal iris disorders, principally iridocorneal endothelial (ICE) syndrome and Axenfeld–Rieger syndrome (ARS). Iridoschisis is diagnosed by slit-lamp examination, which reveals the iris’s characteristic layered separation. ICE syndrome typically affects young adults, with a predilection for women in their 30s and 40s, and is usually unilateral. Diagnostic hallmarks include: corectopia/polycoria, iris atrophy, full-thickness holes, iris nodules, and endothelial dystrophy (11). Axenfeld-Rieger syndrome (ARS) is a bilateral congenital glaucoma, typically diagnosed in adolescence. Its anterior segment abnormalities include posterior embryotoxon, corectopia, iris dysplasia, and iridocorneal adhesions (12). Our patients exhibited findings consistent with iridoschisis.

Iridoschisis is associated with glaucoma in over two-thirds of cases, predominantly angle-closure type (2). When angle closure or significant narrowing occurs, LPI is commonly used to alleviate pupillary block (1). In Case 1, pupillary block was temporarily resolved after LPI. Imaging (UBM/AS-OCT), however, revealed a still shallow anterior chamber and trabecular meshwork obstruction by iris tissue. The patient in Case 2 had iridoschisis and a narrow angle. During right eye surgery, partial anterior synechiae were noted in the angle. We suggest that cataract extraction removes the opaque lens, reduces its anterior chamber occupation, and—when combined with IOL implantation—widens the angle and deepens the anterior chamber. Goniosynechialysis employs a viscoelastic agent to mechanically separate angle synechiae, directly addressing the physical obstruction underlying angle closure. These combined procedures reestablish normal aqueous outflow to lower IOP while also addressing cataract-induced visual impairment. The presence of iridoschisis elevates angle-closure glaucoma risk in anatomically predisposed patients. Our two cases, despite their differing clinical presentations, both illustrated that the combined procedure of phacoemulsification and goniosynechialysis is a safe and feasible approach for iridoschisis accompanied by angle-closure and cataract. Early surgery combining cataract extraction and goniosynechialysis is recommended for expansive cataract with iridoschisis.

Cataract surgery in eyes with iridoschisis poses technical challenges. Loose iris tissue often obstructs the phacoemulsification tip, causing iris damage. While viscoelastic agents can stabilize loose iris tissue (13), and microcautery or vitreous trimming may reduce fibers (14, 15), we excised with Vannas scissors to avoid corneal endothelial contact by floating fibers, which risks decompensation.

In Case 1, the patient was notable for positive treponemal serology in the absence of active ocular inflammation. This pattern suggests a late or cured syphilitic infection, which is a recognized, albeit uncommon, cause of iridoschisis (4, 16). The proposed mechanism involves chronic immunological damage from congenital or late syphilis leading to iris stromal atrophy and cleavage. While no anti-syphilitic treatment was indicated, and the finding did not alter surgical management, this case underscores the value of considering syphilis screening in patients with iridoschisis of uncertain etiology.

## 4 Conclusion

Iridoschisis requires prompt intervention when ocular complications arise. Combined goniosynechialysis and cataract surgery addresses glaucoma in these patients. As a retrospective case report, this study warrants validation through larger cohorts.

## Data Availability

The original contributions presented in the study are included in the article/Supplementary material, further inquiries can be directed to the corresponding author.

## Supplementary material

SUPPLEMENTARY FIGURE S1 Preoperative bilateral fundus OCT images of Case 1.

SUPPLEMENTARY FIGURE S2 Preoperative bilateral visual field images of Case 1.

SUPPLEMENTARY FIGURE S3 Preoperative bilateral fundus OCT images of Case 2.
