# Supplementary figures and images for: Case Report: Combined cataract surgery and goniosynechialysis in elderly patients with iridoschisis—a report of two cases
Source: Front Med (Lausanne). 2026 Jun 29;13:1873711. doi: 10.3389/fmed.2026.1873711 (PMC13357525; doi:10.3389/fmed.2026.1873711)

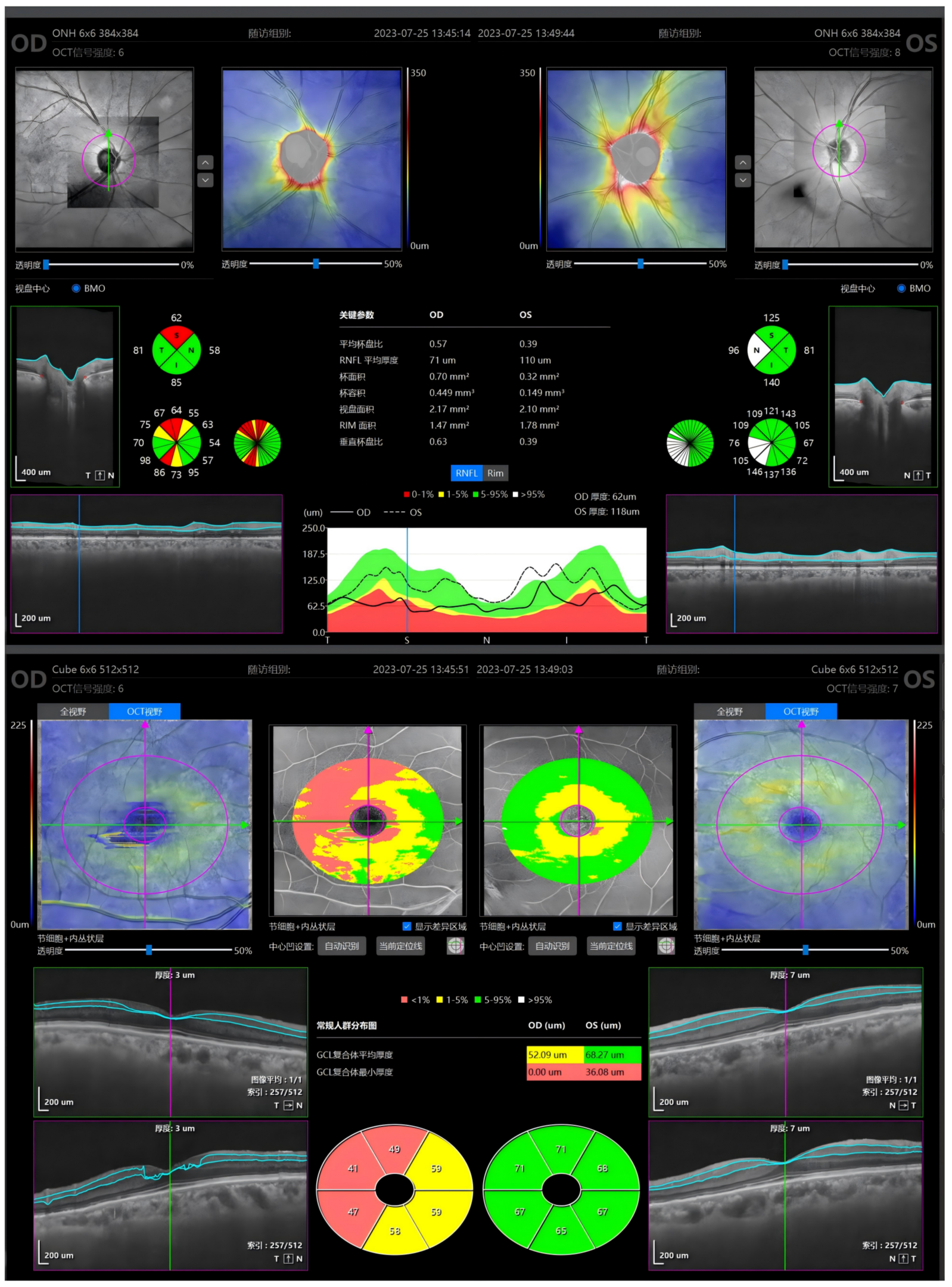

Supplement: SUPPLEMENTARY FIGURE S1 — Preoperative bilateral fundus OCT images of Case 1. [file Image_1.TIFF]

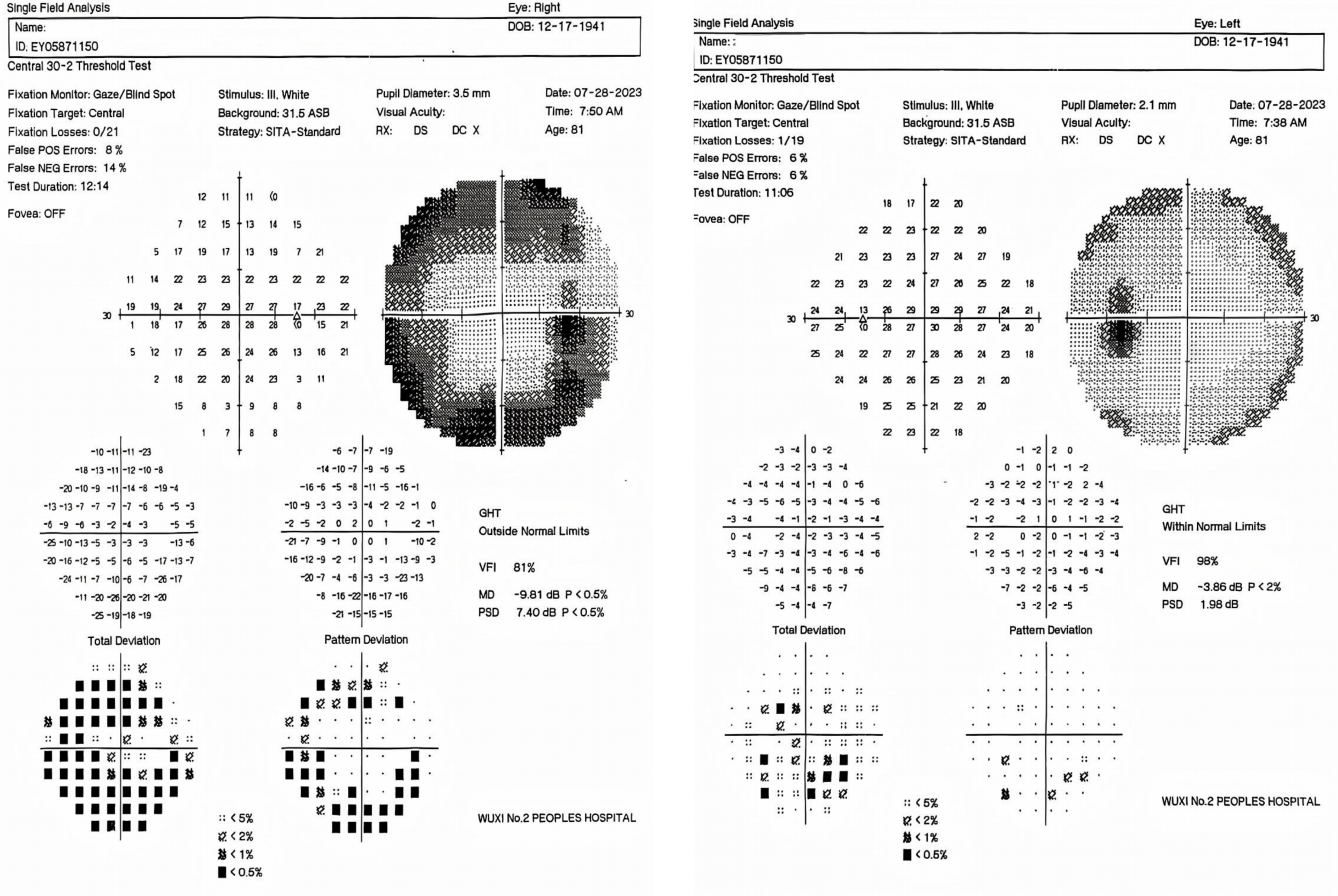

Supplement: SUPPLEMENTARY FIGURE S2 — Preoperative bilateral visual field images of Case 1. [file Image_2.TIFF]

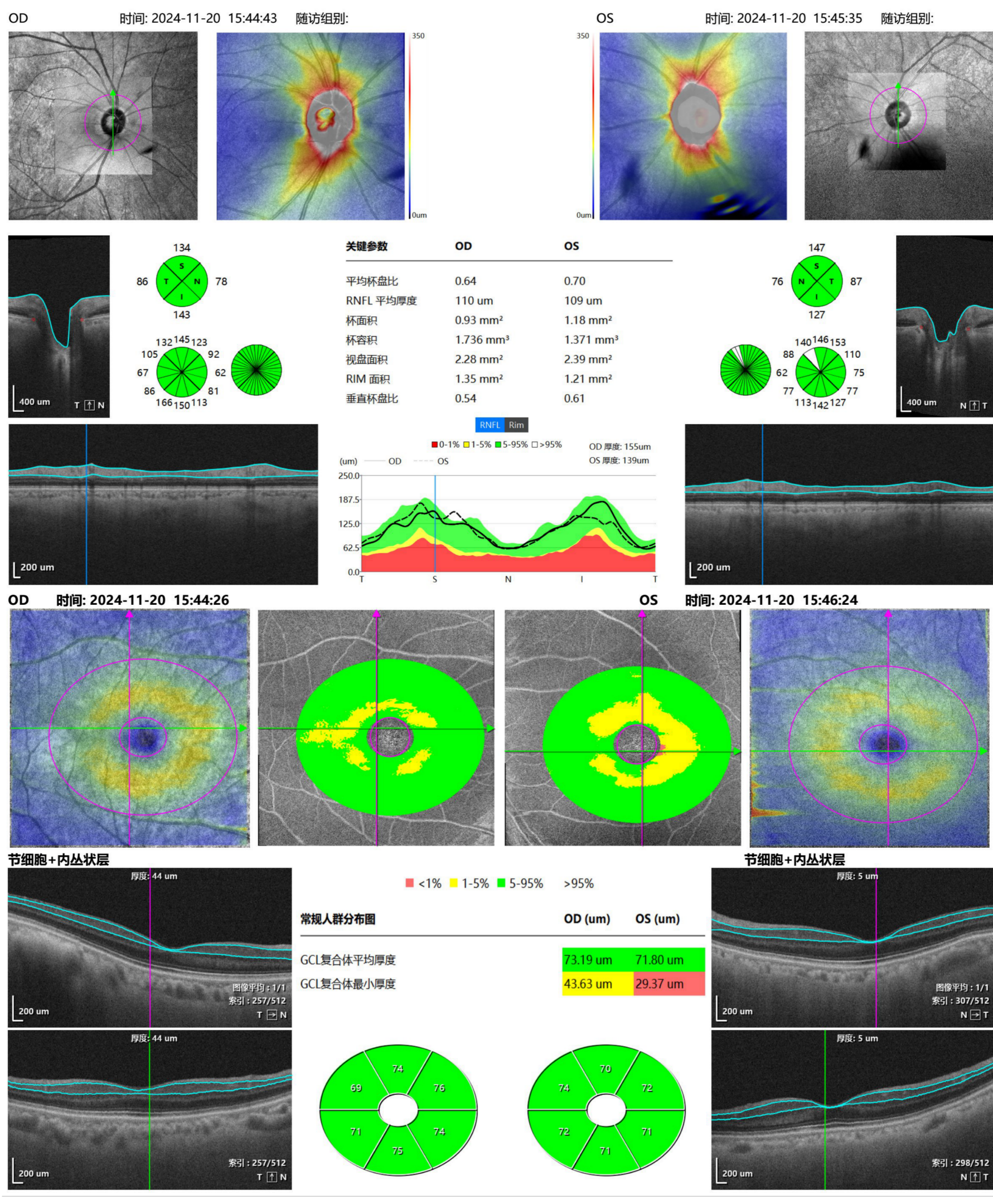

Supplement: SUPPLEMENTARY FIGURE S3 — Preoperative bilateral fundus OCT images of Case 2. [file Image_3.TIFF]
